# Embryonic Senescence and Laminopathies in a Progeroid Zebrafish Model

**DOI:** 10.1371/journal.pone.0017688

**Published:** 2011-03-30

**Authors:** Eriko Koshimizu, Shintaro Imamura, Jie Qi, Jamal Toure, Delgado M. Valdez, Christopher E. Carr, Jun-ichi Hanai, Shuji Kishi

**Affiliations:** 1 Department of Ophthalmology, Harvard Medical School, Boston, Massachusetts, United States of America; 2 Graduate School of Marine Science and Technology, University of Marine Science and Technology, Tokyo, Japan; 3 Department of Earth, Atmospheric and Planetary Sciences, Massachusetts Institute of Technology, Cambridge, Massachusetts, United States of America; 4 Division of Nephrology, Interdisciplinary Medicine and Biotechnology, Department of Medicine, Beth Israel Deaconess Medical Center, Harvard Medical School, Boston, Massachusetts, United States of America; 5 Department of Metabolism and Aging, The Scripps Research Institute, Jupiter, Florida, United States of America; University of Florida, United States of America

## Abstract

**Background:**

Mutations that disrupt the conversion of prelamin A to mature lamin A cause the rare genetic disorder Hutchinson-Gilford progeria syndrome and a group of laminopathies. Our understanding of how A-type lamins function *in vivo* during early vertebrate development through aging remains limited, and would benefit from a suitable experimental model. The zebrafish has proven to be a tractable model organism for studying both development and aging at the molecular genetic level. Zebrafish show an array of senescence symptoms resembling those in humans, which can be targeted to specific aging pathways conserved in vertebrates. However, no zebrafish models bearing human premature senescence currently exist.

**Principal Findings:**

We describe the induction of embryonic senescence and laminopathies in zebrafish harboring disturbed expressions of the lamin A gene (*LMNA*). Impairments in these fish arise in the skin, muscle and adipose tissue, and sometimes in the cartilage. Reduced function of lamin A/C by translational blocking of the *LMNA* gene induced apoptosis, cell-cycle arrest, and craniofacial abnormalities/cartilage defects. By contrast, induced cryptic splicing of *LMNA*, which generates the deletion of 8 amino acid residues lamin A (zlamin A-Δ8), showed embryonic senescence and S-phase accumulation/arrest. Interestingly, the abnormal muscle and lipodystrophic phenotypes were common in both cases. Hence, both decrease-of-function of lamin A/C and gain-of-function of aberrant lamin A protein induced laminopathies that are associated with mesenchymal cell lineages during zebrafish early development. Visualization of individual cells expressing zebrafish progerin (zProgerin/zlamin A-Δ37) fused to green fluorescent protein further revealed misshapen nuclear membrane. A farnesyltransferase inhibitor reduced these nuclear abnormalities and significantly prevented embryonic senescence and muscle fiber damage induced by zProgerin. Importantly, the adult Progerin fish survived and remained fertile with relatively mild phenotypes only, but had shortened lifespan with obvious distortion of body shape.

**Conclusion:**

We generated new zebrafish models for a human premature aging disorder, and further demonstrated the utility for studying laminopathies. Premature aging could also be modeled in zebrafish embryos. This genetic model may thus provide a new platform for future drug screening as well as genetic analyses aimed at identifying modifier genes that influence not only progeria and laminopathies but also other age-associated human diseases common in vertebrates.

## Introduction

The *LMNA* gene encodes A-type lamins, lamin A and lamin C. The lamins are structural protein components of the nuclear lamina and constitute a class of intermediate filaments, which form a mesh network underlying the inner nuclear membrane that determines nuclear shape and size [1] [2] [3] [4]. Mutations in genes encoding the nuclear lamins and associated proteins cause a wide spectrum of human diseases sometimes called “laminopathies.” Diseases caused by mutations in *LMNA* encoding the A-type lamins include more than 10 different clinical syndromes [5]. Major types include diseases of the striated and cardiac muscle, lipodystrophy syndromes, peripheral neuropathies, and premature aging. While mutations and clinical phenotypes of laminopathies have been carefully described, explanations of the underlying pathogenic mechanisms are still emerging. One key question is: how do numbers of different mutations in this single gene produce such phenotypic heterogeneity and so many different diseases.

Heterozygous point mutations in lamin A cause Hutchinson-Gilford progeria syndrome (HGPS) [6] [7]. Most cases of HGPS harbor an identical *de novo* single-base substitution resulting in a silent Gly-to-Gly change at codon 608 within exon 11. This substitution creates an exonic consensus splice donor site/sequence and activates cryptic splicing, leading to the deletion of 50 codons at the end of prelamin A, called “progerin” [8] [9] [10]. The lamin A is synthesized as the precursor molecule prelamin A [11], and the maturation of lamin A involves the removal of 15 residues from the C terminus cleaved by the endoprotease, Zmpste24/FACE1, via isoprenylation and farnesylation involving a C-terminal CAAX (cysteine-aliphatic-aliphatic-any amino acid) box [12]. Progerin retains the CAAX box farnesylated at its C terminus, but lacks the site for endoproteolytic cleavage, and accumulates at the nuclear envelope, causing misshapen nuclei [13]. Importantly, sporadic use of the cryptic splice site in *LMNA* exon 11 has also been demonstrated during the normal human aging [14].

Notably, lethal neonatal laminopathies have now been identified and are caused by some specific mutations in the *LMNA* and *ZMPSTE24*/*FACE-1* genes, suggesting that certain developmental abnormalities may be involved in some cases [15] [16] [17]. Whilst the crucial involvement of lamin A and lamina-associated polypeptide (LAP) 2α in the regulation of stem cells has been suggested previously [18] [19] [20] [21] [22] [23] [24], and several developmental studies have been performed in model animals including *Drosophila*, *Xenopus*, chicken, cow and mouse [25] [26] [27] [28] [29] [30], the essential roles for this gene and its requirement during early vertebrate development are still poorly understood *in vivo*. In addition, several gene perturbations identified by microarray profiling have also now been confirmed in HGPS fibroblasts [31] [32] [33] [34], but very few have been validated extensively in animal models.

Previous studies of HGPS fibroblasts have shown that farnesyltransferase inhibitors (FTIs) reduce the nuclear abnormalities associated with prelamin A accumulation and improve the disease phenotypes in mouse models [35]. More recently, it has been reported that a combined drug treatment with statins and aminobisphosphonates, which efficiently inhibits both farnesylation and geranylgeranylation of progerin and prelamin A, markedly improves the aging-like phenotypes and lifespan of mice deficient in Zmpste24 [36]. These findings suggest that the compounds may provide new therapeutic approaches to the treatment of this devastating progeroid syndrome. However, given the evidence of persistent disease in mice expressing a non-farnesylated version of progerin, FTIs may not represent a “panacea” for this disease [37] [38]. Hence, new therapeutic interventions need to be considered, and new vertebrate model systems that facilitate high-throughput screening of chemical compounds to treat HGPS and other laminopathies are also essential. The zebrafish (*Danio rerio*) system has the potential to emerge as a key vertebrate model in this regard, as zebrafish maintain evolutionally conserved A-type (A and C) as well as B-type (B1 and B2) lamins among vertebrates.

We here describe that two different types of morpholino antisense oligonucleotides (MOs) directed against the zebrafish *LMNA* gene. The first of these MOs (*LMNA*-MO1) introduces a knockdown of zebrafish lamin A/C, and the other MO (*LMNA*-MO2 or -MO2.1) induced an unique cryptic splicing in exon 11 to generate a new truncated form (zebrafish lamin A-Δ8) in the globular domain of zebrafish lamin A. These MOs caused specific disruptions in the development of zebrafish embryos, particularly in the mesenchymal cell lineages, which are accompanied by either excessive apoptosis or embryonic senescence. We further demonstrate that an FTI can efficiently inhibit the farnesylation of zebrafish progerin (zebrafish lamin A-Δ37/zProgerin) and suppress the abnormal nuclear morphologies, muscle fiber defects and embryonic senescence phenotypes. Accelerated aging-like symptoms and shortened longevity were also observed in zebrafish expressing progerin/lamin A-Δ37 or even prelamin A. Our genetic model using zebrafish may thus facilitate a high-throughput approach to the search for novel therapeutics for human progeroid syndromes and laminopathies associated with nuclear-envelope abnormalities.

## Results

### Transgenic Expression of Zebrafish Progerin and Prelamin A

**Figure 1:**
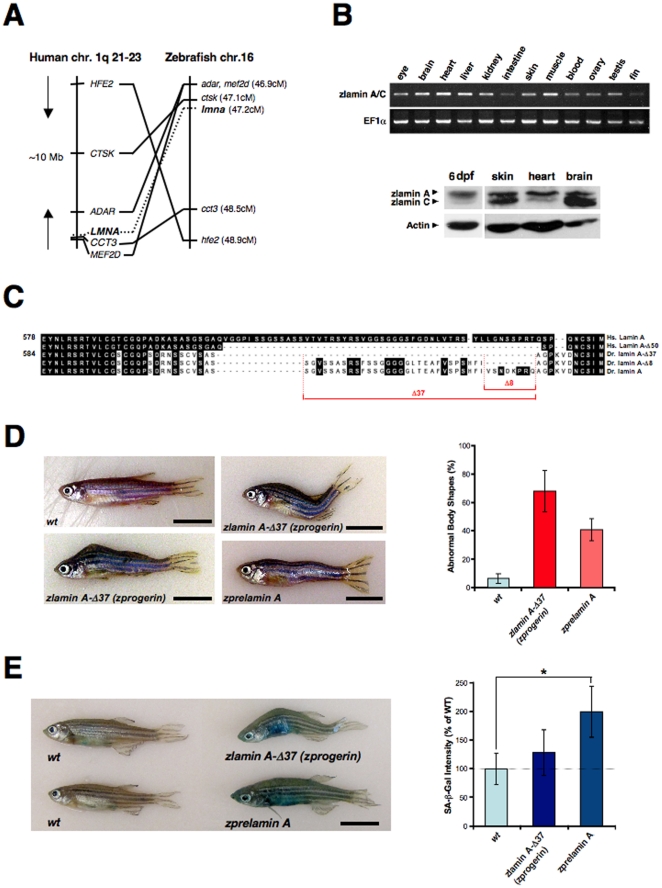
Transgenic expression of zebrafish Progerin/lamin A-Δ37 and prelamin A. (**A**) The zebrafish *LMNA* gene was assigned to Linkage Group 16 (LG16) and is syntenic with human chromosome 1q21-23. Left: six genes including *LMNA* are located within a 10 Mb genomic region on human chromosome 1. Right: six zebrafish homologs of these genes are listed according to the map positions on LG16. (**B**) RT-PCR analysis of adult zlamin A revealing strong expression in the brain, heart, liver and muscle (upper panels). Western analysis of 6 dpf embryo lysates and 1-year old adult tissues was preformed using an anti-chicken lamin A polyclonal antibody (ab14309), as well as an anti-mouse actin monoclonal antibody as a loading control (lower panels). (**C**) Amino acid sequence alignment of the C-terminal portions in human (*Homo sapiens*) lamin A (Hs. lamin A) and zebrafish (*Danio rerio*) lamin A (Dr. lamin A) and their deleted forms (Δ50 in human; Δ37 and Δ8 in zebrafish) induced by cryptic splicing events. (**D**) External features of a wild-type non-transgenic sibling fish (*wt*; 1.5-year old). Two representative *zprogerin* transgenic fish with curved trunks (*zlamin A-Δ37*; 1.5-year old) are shown. A z*prelamin A* transgenic fish with a wavy trunk (2.1-year old) is also shown. In these transgenic fish, spinal curvatures and disproportional body shapes are prominently observed. Scale bars, 1 cm. Qualifications of the abnormal body shapes are shown in the right graph (cohorts of 18 months of age). (**E**) SA-β-gal staining of wild-type sibling and transgenic fish (1.9-year old). Quantifications of the SA-β-gal intensities in the fish are shown in the right graph (cohorts of 18 months of age, *P<0.01).

The zebrafish *LMNA* gene was assigned to Linkage Group 16 (LG16) by radiation hybrid (RH) mapping of the LN54 RH panel [39]. The zebrafish *LMNA* region on LG16 is syntenic with human chromosome 1q21-23, which includes the genes *adar* (*adenosine deaminase, RNA-specific*), *mef2d* (*MADS box transcription enhancer factor 2*), *ctsk* (*cathepsin K*), *cct3* (*chaperonin containing TCP1, subunit 3 (gamma)*), and *hfe2* (*hemochromatosis type 2*) in addition to *LMNA*. This synteny strengthened the validity of the *LMNA* gene location based on RH mapping and physical linkage, and strengthened the identity of zebrafish *LMNA* as an ortholog of human *LMNA*. Upon the molecular cloning of zebrafish *LMNA* cDNA (**Fig. S1**), as well as conserved syntenic mapping analysis between the human and zebrafish *LMNA* genes (**Fig. 1A**), we developed zebrafish models for HGPS and laminopathies. Reverse transcriptase-PCR (RT-PCR) analysis of adult zlamin A/C revealed ubiquitous expression throughout the tissues, but at higher levels in the brain, heart, liver and muscle (**Fig. 1B**). We also examined the protein expression of zlamin A/C in adult tissues by western blotting analysis and found its ubiquitous expression as anticipated, although zlamin A was more strongly expressed in heart and higher levels of zlamin C were evident in brain (**Fig. 1B** **, lower panels**).

As HGPS is a dominant sporadic disease caused by mutations within exon 11 of *LMNA*, leading to an in frame deletion of 50 amino acids from the C-terminal tail of prelamin A (Progerin/lamin A-Δ50), we first attempted to generate transgenic zebrafish expressing a form of zebrafish lamin A with a deletion of 37 amino acids i.e. a zlamin A-Δ37 (zebrafish Progerin: zProgerin) expression construct. This was to closely mimic the effects of human Progerin/lamin A-Δ50 based on the alignment of their amino acid sequence (see **Fig. 1C**). We microinjected wild-type zebrafish embryos with a zProgerin expression construct driven by the human cytomegalovirus (CMV) promoter at the one-cell stage of development for the generation of founder (F_0_) fish that showed mosaic expression of the transgene [40]. In contrast to healthy non-transgenic *wild-type* (*wt*) siblings (**Fig. 1D** **, upper left picture panel**), of the 21 germline-positive F_0_ zebrafish injected with enhanced green fluorescent protein (EGFP)-zProgerin, 19 (90%) were symptomatic for early spinal curvature, wasting symptoms, and odd swimming behaviors in young- to mid-adults (**Fig. 1D** **, lower left and upper right picture panels**), as were 3 (25%) of 12 germline-positive fish injected with EGFP-zebrafish prelamin A (zPrelamin A) (**Fig. 1D** **, lower right picture panel**). The affected transgenic zebrafish developed distended curvatures ranging from a mild bend or arch to a mild or moderate distortion, which are similar to manifestations of senescence reported previously [41]. Some fish had protruding ventral or dorsal curvatures with relatively small pectoral fins, whereas others had wavy trunks throughout (**Fig. 1D**). Many of these fish also showed extensively unbalanced body shapes with age, resulting subsequently in the onset of abnormal swimming behaviors in their later lives. The mean latencies of the phenotype development in *zprogerin* and *zprelamin A* fish groups were 332 (range of 221 to 444 days) and 352 days (range of 306 to 431 days), respectively. All of the germline-positive F_0_ *zprogerin* fish died before reaching 2 years of age. On the other hand, non-symptomatic F_0_ *zprelamin A* fish survived past 3 years although the 3 symptomatic fish died before reaching 2.5 years. In order to determine whether *zprogerin* and *zprelamin A* fish were prematurely aging, we used senescence-associated β-galactosidase (SA-β-gal), a hallmark of cellular senescence *in vitro* as well as a marker of the organismal aging process in vertebrates including zebrafish [42] [43] [44] [45] [46]. Sporadically enhanced SA-β-gal activity was observed in approximately 70% of morphologically still non-symptomatic (almost normal shaped) transgenic fish (one of z*prelamin A* fish with high SA-β-gal is shown; **Fig. 1E** **, left picture panel**). We further confirmed the appearance of these accelerated aging symptoms in another cohort (18 months of age) comprising uninjected *wt* sibling (n = 56), F_0_ *zprogerin* (n = 34), or F_0_ *zprelamin A* fish (n = 28) (**Fig. 1D and E** **, right graphs**). Intriguingly, the frequency of abnormal body shapes was higher in the *zprogerin* transgenic fish group than in the *zprelamin A* fish group, whereas the SA-β-gal intensities were much higher in the *zprelamin A* fish. These results suggest that accelerated aging symptoms may develop in most of the mosaicked transgenic fish whose cells carry a functional *zprogerin* or *zprelamin A* transgene that is sporadically expressed.

### Knockdown of The LMNA Gene in Zebrafish Embryos Showing Muscle and Cartilage Abnormalities

**Figure 2:**
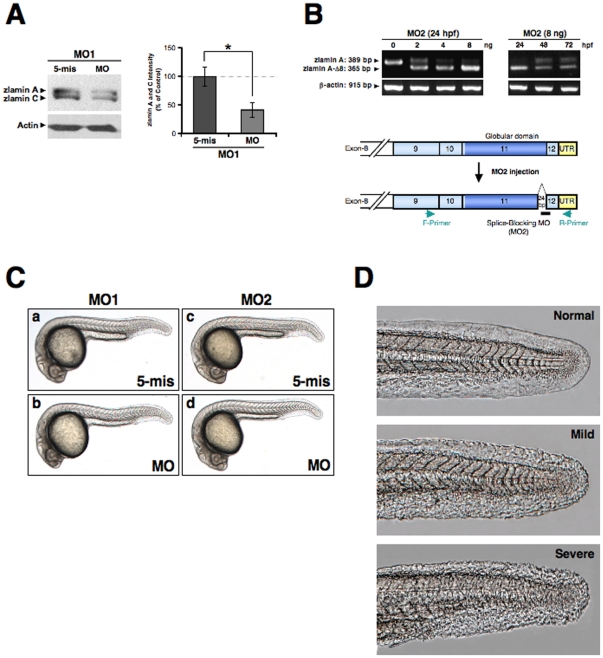
Knockdown of zebrafish lamin A/C. (**A**) Western blot analysis of intrinsic lamin A/C in the zebrafish AB9 cells transfected with MOs. The lower panel of blotting shows the actin control (*P<0.01). The right-side graph shows the quantitation results for the lamin A and C blot intensities standardized by the control actin levels from three-independent experiments. (**B**) Agarose gel electrophoresis of the RT-PCR products of zlamin A from embryos injected with the indicated concentrations of z*LMNA*-MO2. The primers used recognize exon 9 (forward primer; F-Primer) and the 3′-untranslated region (reverse primer; R-Primer). Sequence analysis revealed that the injection of z*LMNA*-MO2 induces a 24 bp deletion in the rear part of exon 11. Left panel, total RNA was extracted from 24 hpf embryos after MO injection. Right panel, embryos were injected with 8 ng MO and total RNA was extracted from 24 to 72 hpf as indicated. Lower panel, the corresponding positions of the F-Primer, R-Primer, and MO2 are schematically presented. (**C**) Gross morphology of zlamin A/C-knockdown embryos. Lateral views of MO-injected embryos (4 ng) at 24 hpf are shown. (a) 5-base mismatch control MO1 (MO1, 5-mis), (b) z*LMNA*-MO1 (MO1, MO), (c) 5-base mismatch control MO2 (MO2, 5-mis), (d) z*LMNA*-MO2 (MO2, MO). (**D**) Occasionally observed rough skin phenotypes (Mild and Severe) in MO2-morphant tails are shown by comparison with a normal skin phenotype (Normal) in the wild-type tail.

**Figure 3:**
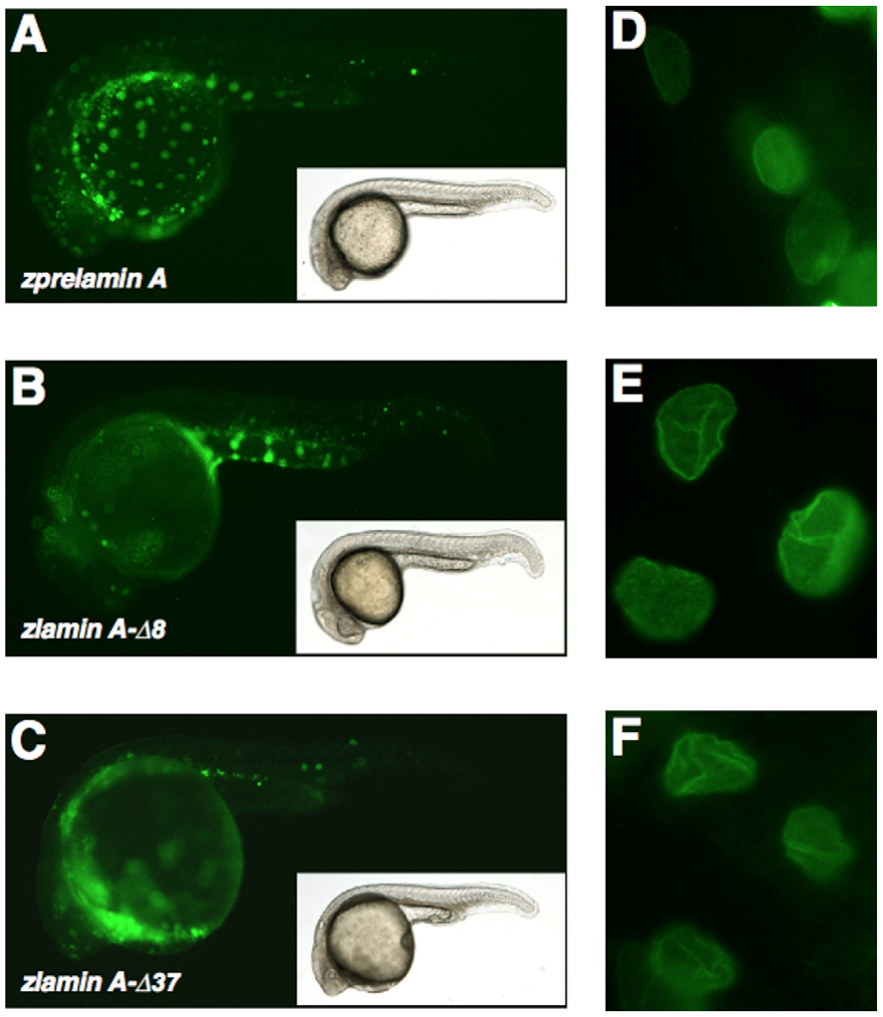
Expression of EGFP-tagged zprelamin A, zlamin A-Δ8, and zlamin A-Δ37/zProgerin in zebrafish embryos and the nuclear morphologies in the expressing cells. (**A**) EGFP-zPrelamin A expression in zebrafish embryos. (**B**) EGFP-zlamin A-Δ8 expression in zebrafish embryos. (**C**) EGFP-zlamin A-Δ37/zProgerin expression in embryos. (**D**) The nuclear localization of EGFP-zPrelamin A and the nuclear morphology in the expressing cells within the embryo. (**E**) The nuclear localization of EGFP-zlamin A-Δ8 and the nuclear morphology in the expressing cells within the embryo. (**F**) The nuclear localization of EGFP-zlamin A-Δ37/zProgerin and nuclear morphology in the expressing cells within the embryo.

Lamin A/C is ubiquitously expressed in multiple tissues from adult zebrafish (**Fig. 1B** **and Fig. S2**), and we have also detected the expression of the corresponding gene, *LMNA*, by whole-mount *in situ* hybridization and semi-quantitative RT-PCR in zebrafish embryos (**Fig. S3**) (**Supplemental Data S1**). Lamin A/C protein expression during zebrafish embryogenesis was found to be relatively lower than that in various adult tissues but this may be an artifact of high yolk protein levels in embryos (**Fig. 1B** **, lower panel as shown for a larval sample at 6 days post fertilization, dpf**). *LMNA*-null mice are born with a normal phenotype [47]. However, it has also been suggested previously that the ectopic expression of prelamin A or exon 9-deleted mutant lamin A affects developmental processes in the early phases of *Xenopus* embryogenesis [26] [48]. To examine the function of the type A lamins in the early development of zebrafish, we first tested translation-block morpholino antisense oligonucleotides (MO) (z*LMNA*-MO1) designed to target the translation initiation codon (ATG) and 5′-untranslated regions of both lamin A and C. To determine the effectiveness of the resulting knockdowns at the protein level, z*LMNA*-MO1-transfected zebrafish AB9 fibroblasts were examined by western blotting. Compared with untransfected cells and control 5-mismatch MO1 (‘5 mis’ for MO1)-transfected cells, a downregulation of lamin A/C protein expression was observed (*P*<0.01) (**Fig. 2A**). We further designed two MOs (z*LMNA*-MO2 and -MO2.1) that target the splicing junction the 3′ end of exon 11 to generate aberrant splicing (**Fig. 2B** and **Fig. S4**). This cryptic splicing of lamin A within exon 11 yields a protein with an 8-amino acid deletion (zlamin A-Δ8) (**Fig. 2B** **and** **Fig. 1C**). This removes the predicted site of Zmpste24/FACE1-mediated endoproteolytic cleavage [49], whereas the actual cleavage site in zPrelamin A has not yet been tested. The EGFP-fused zPrelamin A, zlamin A-Δ8 and zlamin A-Δ37/zProgerin proteins each localized at the nuclear envelope in transgenic embryos (**Fig 3** **, D–F**). The specific localization of the zlamin A-Δ8 and zlamin A-Δ37 proteins at the nuclear envelope showing abnormally wrinkled misshapen structures was concurrently observed in embryos expressing both constructs (**Fig. 3E and F**).

**Figure 4:**
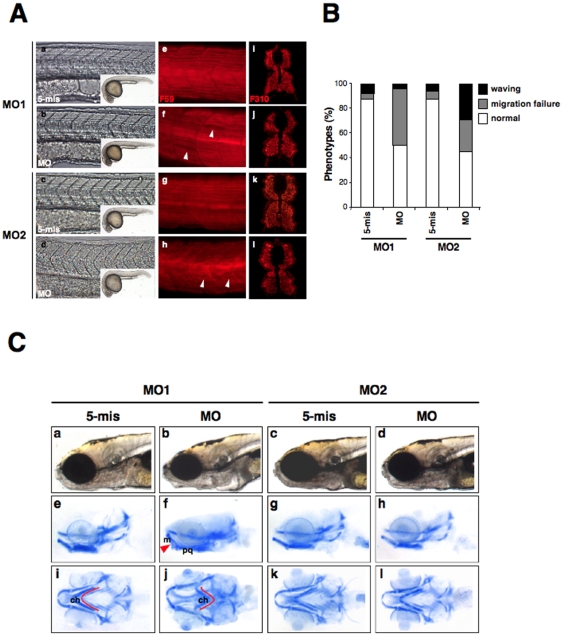
The knockdown of zebrafish lamin A/C induces laminopathies associated with muscular dystrophy and craniofacial abnormalities. (**A**) Muscle fiber visualization in zlamin A/C-knockdown embryos. (a–d) The left panels show a lateral view of the entire embryo bodies and a magnified bright field view of the somites. (e–h) Lateral views of MO-injected embryos at 24 hpf stained with the F59 antibody (adaxial cells/slow muscle fibers). In panel (f), white arrowheads indicate broken or missing muscle fibers. In panel (h), white arrowheads indicate the wavy muscle fibers in compared with control embryos. (i–l) The right panels show cryostat sections of 48 hpf *LMNA*-MO injected embryos stained for fast muscle fibers (F310). (**B**) Percentage of muscle phenotypes scored for MO-injected embryos at 24 hpf. The graph shows the distribution of phenotypes observed following injections with 4 ng of MOs. Migration failure in embryos is represented in gray and wavy muscle fibers in black. The percentage of each muscle phenotypes ‘waving’, ‘migration failure’, ‘normal’ were, respectively, MO1-5mis; 7.5%, 5.0%, 87.5%; MO1-MO; 3.8%, 46.2%, 50.0%; MO2-5mis; 6.1%, 6.1%, 87.8%; MO2-MO; 29.4%, 25.5%, 45.1%. (**C**) Cartilage in 6 dpf larvae were stained with Alcian blue and then whole-mounted. (a–d) Lateral views of the head portions of living larvae are shown in the top panels. (e–h) Lateral view of Alcian blue stained larvae. (i–l) Ventral views of Alcian blue stained larvae. The red lines indicate the ceratohyal articulates (ch), and the red arrow indicates the Meckel's (m) to palatoquadrate (pq).

The phenotype induced by the knockdown of zebrafish lamin A/C by different MOs during early development was not particularly evident at the gross morphology level at 24 hours post fertilization (hpf) (**Fig. 2C** **)**, except an occasional observation of rough skin (**Fig. 2D**). However, when we analyzed slow skeletal muscle fibers in more detail by staining myosin heavy chains with an F59 antibody (**Fig. 4A** **, e–h**), muscle damage was evidenced by a failure in slow muscle fiber migration to the lateral surface along with irregular, gapping (**Fig. 4A** **, f; MO1**), and/or wavy (**Fig. 4A** **, h; MO2**) muscle fibers in approximately 50% of the z*LMNA*-morphants (**Fig. 4B**). In contrast, no evident differences in fast muscle fibers in the morphants were identified via the staining of myosin light chains with an F310 antibody (**Fig. 4A** **, i–l**). We further demonstrated the slow muscle phenotype via expression of the *zprogerin* (*zlamin A-Δ37*) transgene. *zprogerin* transgenic zebrafish embryos (∼40%) showed similar slow muscle fiber abnormalities as shown later.

We next examined craniofacial phenotypes in our morphants, as some mutations in the *LMNA* gene are known to cause craniofacial anomalies and/or disproportion in both humans and mice [50] [51]. In z*LMNA*-MO1 morphants at 6 dpf, a dorsal displacement of Meckel's cartilage in relation to the palatoquadrate (n = 32 of 65; 49%) (red arrow in **Fig. 4C** **, f**) was observed and the jaw appeared to be shorter in the ventral view of the head (**Fig. 4C** **, j**). In contrast, this seemed not to be due to a reduction in the size of any elements of the jaw apparatus but rather to a reduced angle between the Meckel's cartilage and the palatoquadrate. Moreover, from the ventral view, retrognathic pharyngeal cartilage defects (a red line in **Fig. 4C** **, j**) were identified in the ceratohyal articulates (a further posterior position than the lateral view) (n = 18 of 65; 28%) (**Fig. 4C** **, j**) (**Table 1**). Although obvious pharyngeal cartilage defects in morphology, including retrognathic formation, were not apparent within z*LMNA*-MO2 morphants (**Table 1**), the MO2 molecule induced a relatively hypoplastic formation of cartilage matrix with occasionally reduced Alcian blue staining, indicating a possible alteration of the composition of the matrix.

**Table 1:** Cartilage defects in LMNA-MO-injected embryos.

	Injection	The Number of Injected Embryos	Ceratohyal Defect
**zLMNA-MO1**	5-mis	100	0% (0/89)
	MO	100	28% (18/65)
**zLMNA-MO2**	5-mis	100	0% (0/82)
	MO	100	0% (0/78)

### Apoptosis, Senescence, and Lipodystrophy in zLMNA Morphants

**Figure 5:**
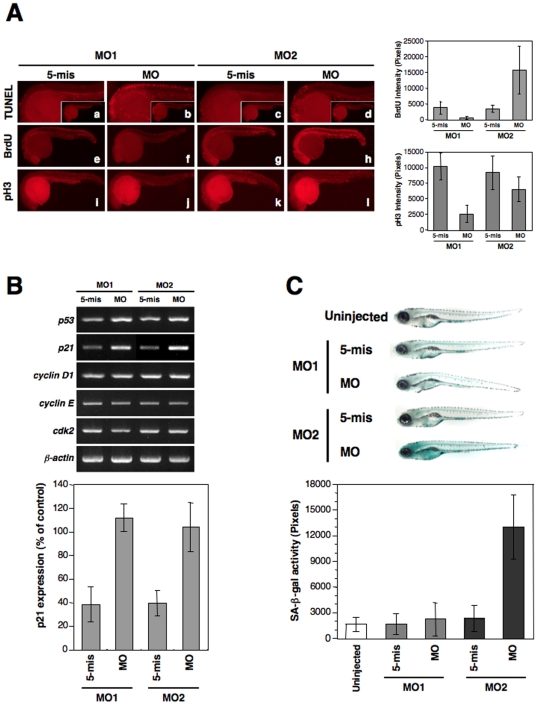
The knockdown of zebrafish lamin A/C induces an aberrant cell cycle, apoptosis, and senescence. (**A**) Apoptosis and cell cycle analysis in 24 hpf embryos. (a–d) Lateral view of apoptotic phenotypes in MO-injected embryos using a TUNEL assay (TUNEL-positive cells are indicated by the red fluorescence spots). z*LMNA*-MO1-injected embryos shows increased apoptosis in the head and trunk. (e–h) BrdU incorporation in MO-injected embryos. z*LMNA*-MO2-injected embryos show increased BrdU-positive cells throughout the whole body. (i–l) MO-injected embryos stained with anti-phospho histone H3 (pH 3). A z*LMNA*-MO1-injected embryo shows a decreased number of pH 3-positive cells. Quantifications of the BrdU and pH 3 intensities in morphants are shown in right graphs (BrdU: MO1, *P*<0.05 for 5-mis versus MO, MO2, *P*<0.05 for 5-mis versus MO; pH 3: MO1, *P*<0.05 for 5-mis versus MO, MO2, no significance for 5-mis versus MO). (**B**) Genes involved in the p53-related and cdk2 pathways were analyzed by semi-quantitative RT-PCR in MO-injected embryos at 24 hpf. Quantifications of the bands intensities for p21 are shown in the bottom graph (*P*<0.01 for 5-mis versus MO in MO1 and MO2). (**C**) SA-β-gal assay of 6 dpf MO-injected larvae in comparison with each control 5-mis MO. Qualifications of the SA-β-gal activity are shown in bottom graph. The z*LMNA*-MO2-injected embryos revealed strong induction of SA-β-gal activity (*P*<0.01 for 5-mis versus MO in MO2), whereas the z*LMNA*-MO1-injected embryos did not exhibit any obvious activity, compared with Cont-MO1-injected embryos.

Given the morphologies observed in our zebrafish morphants, we assayed for apoptotic cell death in these embryos using the TUNEL assay. Apoptotic cells were predominantly detected in the brain and trunk of the z*LMNA*-MO1-injected embryos at 24 hpf (**Fig. 5A** **, b**). On the other hand, in z*LMNA*-MO2-injected embryos, we observed only a slight induction of apoptosis (**Fig. 5A** **, d**). In the control embryos, no significant apoptosis induction was observed (**Fig. 5A** **, a, c**), although scattered apoptotic cells were occasionally detectable at background levels throughout the embryos.

Given the obvious appearance of apoptotic cells in our morphants, we assessed whether cell proliferation was altered in any way. Although the expression of proliferating nuclear antigen (PCNA) was not substantially affected (**Fig. S5**), the cell-cycle progression was prominently disturbed in the morphants. Progression through the S and M phases of the cell cycle, assessed by the incorporation of 5-bromo-2-deoxyuridine (BrdU) and the immunostaining of a mitotic marker, phosphorylated histone H3 (pH 3), respectively, was decreased in both cases in the z*LMNA*-MO1 morphants (**Fig. 5A** **, f, j, see right graph**). Intriguingly, in the z*LMNA*-MO2 morphants, an accelerated progression through S phase was observed but no significant alterations during M phase were detected (**Fig. 5A** **, h, l, see right graph**), implicating the accumulation/arrest in S-phase with increased DNA damage and/or dysregulated DNA repair [52].

Semi-quantitative RT-PCR was used to assay individual morpholino-injected embryos for the expression of *p53*, *p21^WAF1/CIP1^*, *cyclin D1*, *cyclin E*, and *cdk2*, with *β-actin* used as a normalization control. A slight upregulation of *p53* mRNA was observed in z*LMNA* morphants (either MO1 or MO2), when compared with wild-type embryos (data not shown) and control 5-mismatch MO-injected embryos (**Fig. 5B**). In addition, a downstream target of the p53 pathway, cyclin-dependent kinase inhibitor 1, *p21^WAF1/CIP1^*, was also correspondingly upregulated in the z*LMNA* morphants (**Fig. 5B**). Interestingly, neither *cyclin D1* nor *cyclin E*, which are required for progression into G1 and through to the start of S phase, showed any transcriptional changes in the morphants and *cdk2*, which associates with *cyclin E* and *cyclin A* during the transition from G1 to S phase, was slightly decreased only in z*LMNA*-MO1-injected embryos, suggesting a specific cell-cycle delay resulting from a total lamin A/C decrease. These results are representative of similar analyses on additional single embryos (wild-type, n = 4; MO1-5mis, n = 4; MO1-MO, n = 8; MO2-5mis, n = 4; MO2-MO, n = 8; data not shown) and suggest that, in the reduced lamin A/C expression (by MO1) or a disrupted splicing of this gene (by MO2), p21^WAF1/CIP1^ is commonly activated. However, the reason for the observed distinct increases in TUNEL-positive apoptotic cells as well as differential cell-cycle profiles remains unclear.

Recently, we have demonstrated that an embryonic (larval) senescence phenotype caused by specific gene mutations and by stress is readily detectable via the SA-β-gal staining of zebrafish embryos and larva within 6 days post-fertilization (dpf) [42]. We therefore examined the zlamin A/C-depletion (by MO1) or zlamin A-disturbance (by MO2) effects in our morphants by staining with SA-β-gal. Intriguingly, the z*LMNA*-MO2 morphants showed a high intensity of SA-β-gal at 6 dpf, whereas z*LMNA*-MO1 morphants did not show significantly detectable SA-β-gal activity (**Fig. 5C**).

**Figure 6:**
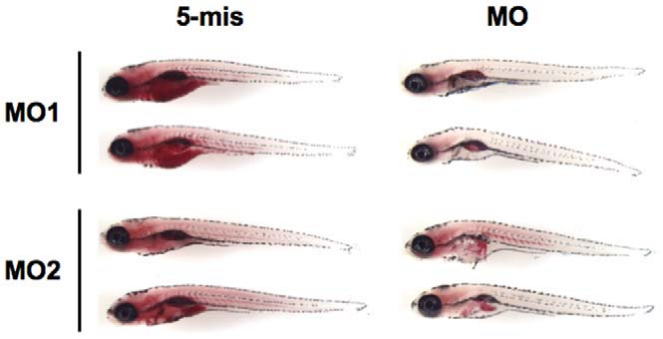
A lipodystrophic phenotype is caused by the zebrafish lamin A/C knockdown. Lateral view of 5 dpf larvae stained with Oil Red O. Strong staining can be observed around the head, the otic vesicle, the jaw, the swim bladder and the heart, as well as in the yolk of the Cont-MO-injected larvae. In contrast, in z*LMNA*-MOs-injected embryos, obvious defects in lipid deposits can be observed in all the tissues except the swim bladder. Duplicate samples are shown for each MO-injected specimen.

Lipodystrophy is one of the hallmarks of the laminopathies and HGPS. To thus examine whether neutral fat distribution is affected in our current morphants, we stained whole larvae with Oil Red O (ORO). Significant ORO lipid staining was detectable in the control larvae as reported previously [53] [54] [55]. However, in the z*LMNA* morphants, a strikingly lower level of lipids was observed in the head, otic vesicle jaw, and yolk (**Fig. 6**). MO1 was found to induce a more severe lipid loss although MO2 still caused a significant lowering of these levels also. To determine the expression levels of zebrafish *peroxisome proliferator-activated receptor-γ* (*PPARγ*), relative to *β-actin* as a control, RT-PCR was performed on individual morpholino-injected embryos. The result showed that either a lamin A/C knockdown by MO1 or the induction of cryptic splicing by MO2 is associated with decreased *PPARγ* mRNA levels (**Fig. S6**), indicating reduced adipogenic differentiation in embryos with lipodystrophic phenotypes.

### FTI Treatment of Transgenic Zebrafish Expressing Progerin

**Figure 7:**
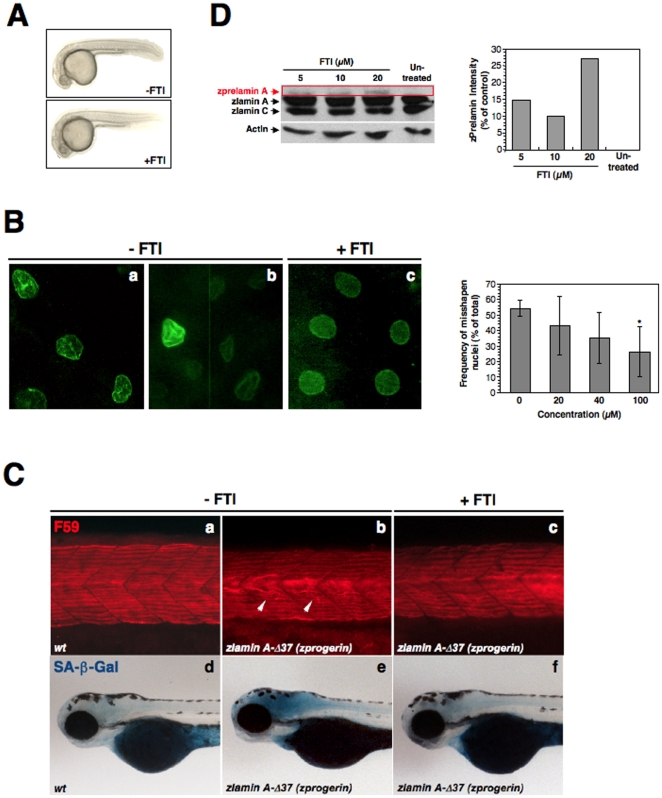
FTI treatment of z*progerin/zlamin A-Δ37* transgenic zebrafish. (**A**) External features of z*progerin* transgenic zebrafish (30 hpf) without (Control, -FTI) or with FTI treatment (100 µM, +FTI). (**B**) Amelioration of nuclear morphology by FTI treatment (100 µM, +FTI) in comparison with untreated (-FTI) z*progerin* fish. Analysis of the misshapen nuclear morphology was also performed by using different concentrations of FTI in z*progerin* transgenic embryos at 30 hpf as shown in right graph. The number of cells with abnormally shaped nuclei were determined by fluorescence microscopy (*P<0.05). (**C**) Amelioration of muscle phenotype (stained with the F59 antibody) at 24 hpf and reduction of SA-β-gal activity at 3.5 dpf in z*progerin* fish embryos by FTI treatment (100 µM, +FTI) in comparison with the untreated condition (-FTI). (**D**) Western blot analysis of prelamin A accumulations in AB9 zebrafish fibroblasts following FTI treatment, and the quantitation of prelamin A amounts in right graph. The quantifications of intrinsic prelamin A accumulations were shown are the averages from two-independent experiments.

To establish the extent of the embryonic phenotypes that arise in lamin A-depleted zebrafish, we further studied the F_1_ generations of germline-positive *zprogerin* transgenic fish since their F_0_ parents showed normal reproduction up until severe aging-like manifestations appeared (**Fig. 1D and E**). Most of the *zprogerin* transgenic fish embryos were morphologically normal, similar to the morphants at 24–30 hpf (**Fig. 7A**). However, all of the examined transgenic fish had the misshapen nuclei (**Fig. 7B** **, a, b**). Once we analyzed their slow skeletal muscle fibers, striking abnormalities with wavy migrations could be observed in a number of cases at 24 hpf (**Fig. 7C** **, b**). The *zprogerin* transgenic fish also showed obvious SA-β-gal induction even at 3.5 dpf (**Fig. 7C** **, e**)

To demonstrate the effects of FTI in zebrafish, we then employed a zebrafish fibroblast cell line (AB9). In FTI treated zebrafish cells, the accumulation of prelamin A was detectable in 20 µM, suggesting that the farnesylation of intrinsic prelamin A was prevented as was further processing of the protein (**Fig. 7D** **, right graph**).

In *zprogerin* transgenic embryos, the ratio of nuclei that had abnormal shapes was 54.3±4.9% (**Fig. 7B**). Previous studies have shown that when the farnesylation of prelamin A in HGPS cells and in Zmpste24-deficient cells is blocked by FTI, prelamin A does not accumulate at the nuclear rim and the percentage of cells with misshapen nuclei is reduced [56] [10] [57] [58] [8] [9]. To determine whether the nuclear shape abnormalities caused by zProgerin in our transgenic zebrafish embryos could also be ameliorated by FTI, different concentrations of this inhibitor were administrated to 5 hpf embryos which were further incubated for 25 h. As expected, the FTI treatment of *zprogerin* transgenic embryos reduced the extent of the abnormally misshapen nuclear morphology in a dose-dependent manner (**Fig. 7B** **, right graph**). The frequency of misshapen nuclei expressing zProgerin (54.3±4.9% in untreated condition) was reduced to 26.3±16.2% upon exposure to 100 µM FTI (*P*<0.05). Moreover, the slow muscle fiber abnormalities, in addition to the onset of embryonic senescence, in these transgenic embryos were significantly blocked by FTI treatment (**Fig. 7C** **, c, f**). These findings are consistent with the results in other progeria cells and animals treated with FTI [59].

### Shortened Lifespan Resulting from Stable Progerin and Prelamin A Expression in Zebrafish

**Figure 8:**
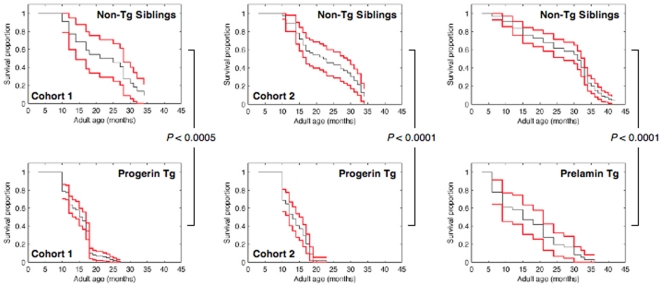
Demographic analysis of the lifespan in *zprogerin* and *zprelamin A* transgenic zebrafish. A shorter lifespan in z*progerin* and z*prelamin A* transgenic zebrafish is demonstrated by Kaplan-Meier survival analysis. In z*progerin* transgenic zebrafish (Progerin Tg), two independent cohorts (Cohort 1 and 2) were analyzed and the survival curves show significant decreases in lifespan compared with the corresponding wild-type siblings (Non-Tg siblings). z*Prelamin A* transgenic zebrafish (Prelamin Tg) also showed a relatively shorter lifespan than their siblings (Non-Tg siblings).

To measure lifespan, stable *zprogerin* or *zprelamin A* transgenic fish were maintained with their wild-type siblings and their survival was measured using Kaplan-Meier survival curves (**Fig. 8**). These fish were not sterile as described above, but their observed lifespan was significantly shorter than that of wild-type zebrafish reported previously [41] [42]. We also examined the *prelamin A* transgenic zebrafish and interestingly, these transgenic fish also suffered from a relatively short lifespan. We maintained populations of these transgenic zebrafish and their wild-type siblings until death, and conducted observational studies on their lifespan. We scored the two cohorts of *zprogerin* transgenic fish (no genders identified; Cohort 1, n = 102; Cohort 2, n = 54) in comparison with their wild-type siblings (no genders identified; Cohort 1, n = 22 and Cohort 2, n = 46), and found significant decreases in the lifespan of the *zprogerin* transgenic animals (Cohort 1, *P*<0.0005; Cohort 2, *P*<0.0001, log rank test) (**Fig. 8**). The plots in **Figure 8** show Kaplan-Meier survival curves and 95 percentile confidence intervals on the cumulative probability distribution (the survival function).

We additionally found that our *zprelamin* A fish cohorts (no genders identified; n = 36) manifested a shorter lifespan compared with that of their wild-type siblings (no genders identified; n = 91; *P*<0.0001, log rank test) (**Fig. 8**). In both the *zprogerin* and *zprelamin A* cases, there were significant differences found between the wild-type siblings and the transgenic fish. These lifespan analyses of *zprogerin* and *zprelamin A* transgenic zebrafish in accordance with the embryonic senescence phenotypes suggest that early developmental manifestations can predict a role for the *LMNA* gene.

## Discussion

The zebrafish is a valuable vertebrate model system for the elucidation of novel molecular pathways of aging as well as early development [60]. In particular, this model organism develops an array of senescence symptoms resembling those in humans [61] [62] [63] [44], and is amenable to large-scale genetic screens that can be targeted to conserved aging pathways in vertebrates [42]. However, the establishment of human disease-associated premature senescence-bearing fish had not been pursued previously. In this study, we developed zebrafish progeria and laminopathy models in three different ways: i) exogenous expression of the zebrafish version of ‘progerin’ (zProgerin) protein, ii) translational-block MO (z*LMNA*-MO1)-induced translational downregulation to reduce lamin A/C protein levels, and iii) splice-block MO (z*LMNA*-MO2)-induced cryptic splicing of *LMNA*, which deleted 8 amino acid residues containing the predicted site of post-translational cleavage. First, the exogenous transgenic expression of zProgerin/zlamin A-Δ37, for which anticipated similar symptoms to MO2 morphants were anticipated, demonstrated a shortened lifespan as well as accelerated aging in adult zebrafish. Second, the translational-block of *LMNA* by MO1 induced apoptosis, cell-cycle arrest, and craniofacial abnormalities (cartilage defects). In contrast, splice-block generating zlamin A-Δ8 with a cryptic splicing of *LMNA* by MO2 produced embryonic senescence and S-phase accumulation/arrest. Interestingly, a lipodystrophic phenotype was induced by both MO1 and MO2. These phenotypic outcomes are summarized in **Table 2** with reference to human progeria and laminopathies. Our data also demonstrated that long-term increased levels of not only progerin but prelamin A in transgenic zebrafish results in a shortened lifespan in these animals. Hence, the expression of normal prelamin A, as well as progerin [14] [64], may be linked to senescence induction during the physiological aging process [65] [66].

**Table 2:** Summary of distinct zebrafish models for laminopathy in this study.

	zLamin A-Δ37 (zProgerin)	zLamin A-Δ8	zPrelamin A	zLMNA-MO1	zLMNA-MO2 zLMNA-MO2.1
**Approach**	Transgenic expression	Transgenic expression	Transgenic expression	Transgenic expression	Splice block of zLMNA in Exon 11 (to generate zLamin A-Δ8)
**Nuclear** **Morphology**	Misshapen	Misshapen	Almost normal, occasionally misshapen	N.A.	N.A.
**Gross Body Morphology**	Almost normal (in embryos) Early body curvature (in adults)	Almost normal (in embryos)	Almost normal (in embryos) Early body curvature (in adults)	Almost normal (in embryos)	Almost normal (in embryos)
**Skin** **Phenotype**	Rough (in embryos)	N.A.	N.A.	Rough (in embryos)	Rough (in embryos)
**Muscle Phenotype** (F59)*	Deficit in smooth muscle (in embryos)	N.A.	N.A.	Deficit in smooth muscle (in embryos)	Deficit in smooth muscle (in embryos)
**Craniofacial Phenotype** (Alcian blue)**	N.A.	N.A.	N.A.	Deficit in cartilage (in embryos)	Normal (in embryos)
**Lipid** (Oil red O)**	N.A.	N.A.	N.A.	Deficit: Decreased lipid; Decreased PPARγ (in embryos)	Deficit: Decreased lipid; Decreased PPARγ (in embryos)
**Apoptosis** (TUNEL)***	N.A.	N.A.	N.A.	Induced (in embryos)	Weakly induced (in embryos)
**Cell Cycle** (BrdU, pH 3)*	N.A.	N.A.	N.A.	Deficit: Decreased S phase; Decreased M phase (in embryos)	Deficit: Increased S phase; Decreased M phase (in embryos)
**Senescence** (SA-β-gal)***	Induced (in embryos) Weakly induced (in adults)	N.A.	Induced (in adults)	Not detectable (in embryos)	Induced (in embryos)
**Lifespan**	Shortened	N.A.	Shortened	N.A.	N.A.

N.A.: not applied or analyzed in this study.

*Antibody-mediated detection.

**Dye-mediated detection.

***Phenotype-specific assay.

Lamin A/C expression is closely correlated with cell differentiation in metazoans, and *LMNA* mRNA is also developmentally expressed in zebrafish embryos from at least the late gastrula period (9 hpf). Some interesting observations were recently reported for developmentally functional A-type lamins in a comparative study of *Drosophila* and humans [25] [67]. On the other hand, detailed insights into any functional role of lamin A/C during early vertebrate development have not been obtained to any great extent to date. Because embryonic stem cells appear to express lamin A/C upon differentiation [20], and lamin A-associated stem cell dysfunction has been linked to disruption of the Wnt and Notch signaling pathways [19] [23] [18] [48], which are essentially involved in the process of early vertebrate development, our current observations of embryonic senescence or apoptosis in *LMNA*-disturbed zebrafish might be consistent with the dysregulation of somatic stem cells leading to organismal premature aging. In particular, it is plausible that the loss/decrease of plasticity and/or onset of self-renewal defects in mesenchymal stem cells may be responsible for the specificity of accelerated tissue degeneration, as most affected tissues in progeria are of mesenchymal origin [18].

Between the knockdown of the *LMNA* gene (by z*LMNA*-MO1) and the induction of mis-splicing within exon 11 of *LMNA* (by z*LMNA*-MO2), some phenotypic differences were observed in treated zebrafish embryos in terms of cell-cycle regulation, apoptosis and senescence. These may be attributable to multiple interacting proteins associated with the cell cycle, including cyclin D3, retinoblastoma protein (pRb), and PCNA [68] [69] [70] [71] [72] [73], although further investigations will be required in this regard. Clearly, there are some specific cell-cycle alterations due to manipulation of lamin A. The knockdown of A-type lamins by MO1 resulted in an arrest of the cell cycle via both an S and M phase block. On the other hand, induced cryptic splicing within exon 11 by MO2 led to increased S phase entry/accumulation without largely affecting M phase to any greater extent. Any linkage of these cell-cycle regulatory mechanisms to apoptosis versus senescence will need further extensive analyses. In this regard, with the exception of *p21^WAF1/CIP1^*, no remarkable differences in the mRNA levels of the cyclins and cdks were observed in our current experiments. As a cyclin-dependent kinase inhibitor, p21 binds and inactivates cyclin-cdk2 or cdk4 complexes including cyclin D3/cdk4 which has been shown to be responsible for the phosphorylation of pRb [74]. p21 also interacts with PCNA and blocks its ability to activate S phase DNA replication and DNA damage repair [74]. Presumably at the inner nuclear membrane, lamin A orchestrates a functional network of the three interacting proteins, cyclin D3, pRb and PCNA under the control of p21 in cell cycle. In addition to the cell-cycle control, p21 can play either anti- or pro-apoptotic roles in cells and also mediate cellular senescence [74]. Hence, it is not entirely surprising that the decrease-of-function of lamin A/C by MO1 and the gain-of-function of zlamin A-Δ8 by MO2 could have such distinct effects on cell cycle, apoptosis and senescence in zebrafish embryos. Since further global gene expression changes as well as epigenetic alterations would be anticipated upon the disruption of lamin A functions [75] [76], the organization and modification of naive chromatin might be worth consideration in future studies.

The phenocopies observed in several zebrafish tissues such as muscle, adipose and skin with their short lifespan as found in human progeria and laminopathies are extremely intriguing. As for the *LMNA* function in our zebrafish progeria model, it should be noted that the decrease-of-function of *LMNA* showed specific developmental defects without any significant induction of SA-β-gal i.e. embryonic senescence. By contrast, specific gain(s)-of-function(s) of farnesylated forms of progerin and prelamin A could play a crucial role(s) in the pathogenesis of the fish progeroid (senescence) phenotype which was reduced by FTI at least in zProgerin embryos. Therefore, the farnesylated progerin and an excess of farnesylated prelamin A may essentially become pathogenic and induce a progeroid phenotype in addition to other laminopathy phenotypes. This would be consistent with the recent observation that exclusive expression of farnesylatable progerin elicited a relatively more severe progeroid phenotypes than those of its non-farnesylated form in knock-in mice [37]. On the other hand, exclusively expressed non-farnesylated prelamin A still induced prominent cardiomyopathy but not progeria in knock-in mice, whereas the survival of such mice which exclusively produce farnesylatable prelamin A was very similar to that of wild-type mice [38], which is quite possibly due to the lower amount of farnesylated prelamin A. Embryonic senescence and further unforeseen developmental abnormalities may also be occurring in human (or mice) progeria given that severe neonatal lethality has been reported [15] [77] [78].

The power of the zebrafish model lies in its ability to accommodate genetic screens to identify responsible or modifier genes and also facilitate compound screens to identify new chemicals that influence developmental processes and signaling pathways. In our current study, we used this tractable system to analyze both embryonic and adult premature senescence, which we modeled on severe pediatric progeroid disease in humans. By generating the stable transgenic zebrafish lines that expresses zProgerin as well as by transiently manipulating the *LMNA*-MOs in early developmental stages of zebrafish embryos, premature aging can now be assumed to be adequately modeled in this system. The fairly late onset of progeria after germline transmission of the transgene made it easier to propagate this stable line. Of note, however, later than fourth generations of transgenic lines onwards showed a gradual decrease in the expression of GFP-zProgerin and no longer maintained a steady-state expression of the transgene further over the multiple generations. Technology to render higher expressing transgenic *progerin* alleles via induction would also be very valuable for *in vivo* use in the zebrafish, and we are currently testing this approach for its capacity to generate large numbers of different vector-based and promoter-driven transgenic fish for subsequently stable genetic manipulations.

The utility of zebrafish to perform screens for small molecules that perturb or restore normal development has been demonstrated [79] [80]. Our reverse-genetic progeroid zebrafish model may also provide the opportunity to conduct large-scale screens for new drugs for the prevention and treatment of normal aging and age-associated diseases in addition to progeria and laminopathies.

## Materials and Methods

### Zebrafish maintenance and ethics

Zebrafish were maintained with a 14∶10 h light/dark cycle and fed living brine shrimp twice daily. Brine shrimp were given using 1 mL pipettes at an amount of about 0.75 mL brine shrimp per 20 fish. Flake food was also given every other day semiquantitatively according to the number of fish in the tanks. A continuously cycling Aquatic Habitats™ system was used to maintain water quality (Apopka, FL) which completely replaces the water in each tank every 6–10 min. Each tank used was a baffle/tank system that eggs water in a circular motion to ensure flushing and water turnover. Ultraviolet (UV) sterilizers (110,000 µW-s cm^−2^) were employed to disinfect the water and prevent the spread of disease in the recirculating system. The water temperature was maintained at 28±0.5°C. The system continuously circulated water from the tanks through Siporax™ strainers, through a fiber mechanical filtration system (three different pore-sized filters; one pre-pad filter of 120 µm, two bag filters of 100 and 50 µm, respectively), and finally into a chamber containing foam filters and activated carbon inserts. Water quality was tested daily for chlorine, ammonia, pH, nitrate and conductivity, and also was under real-time computer monitoring with alarms to signal potential fluctuations. The general health of each fish was also observed on a daily basis, and abnormal looking or acting fish were quarantined into isolated tanks that were not connected to the general circulation. The water in these quarantined tanks was treated with methylene blue. If and when fish recovered, they were returned to their original tanks on the general circulation system. The zebrafish embryos were staged by hours post fertilization (hpf) at 28.5°C and also by morphological criteria for experiments [81].

All animal experiments were approved by and conducted in accordance with the guidelines established by the Institutional Animal Care and Use Committee (IACUC) at The Scripps Research Institute, IACUC approval number 09-0009 and The Schepens Eye Research Institute, IACUC approval number S-150-0909 and S-186-1210.

### Molecular cloning of zebrafish lamin A/C

To isolate the full length zebrafish lamin A/C cDNA, the zebrafish lamin A mRNA sequence deposited in GenBank was used (accession number AF397016) [82]. mRNA was isolated from zebrafish embryos at 24 hpf using QuickPrep mRNA purification Kit (Amersham Biosciences, Uppsala, Sweden). Poly(A) RNA was then purified with oligo(dT)-resin. Double-stranded cDNA was synthesized using M-MLV reverse transcriptase (Promega, Madison, WI), followed by PCR by Ex*Taq* (Takara Mirus Bio, Madison, WI). The primers used were as follows: forward (F): 5′-ATGGAGACTCCAGGTCAGAAAC-3′, reverse (R): 5′-AAGCACAGATGGTCAGGGTTTG-3′. The PCR program entailed a 30-cycle amplification for 30 sec at 94°C, 30 sec at 60°C, and 4 min at 72°C. The amplified DNA fragments were purified by excision from an agarose gel, and cloned into the pCRII-TOPO cloning vector (Invitrogen, Carlsbad, CA). Nucleotide sequences were determined with an automated sequencer (ABI 3700, AME Bioscience, Norway) using the ABI BigDye V3.1 cycle sequencing system.

To analyze the full-length sequence of the zebrafish lamin C gene, 3′- rapid amplification of cDNA ends (3′-RACE) was performed with a Generacer kit (Invitrogen). A forward primer (5′-CAGAACTCATGGGGCAGCGGTGATTTGT-3′; residues 535-544 of zebrafish lamin A) and a forward nested primer (5′-GCGGTGATTTGTTCCAGACCACCCTCAT-3′; residues 540–549 of zebrafish lamin A) were used to amplify the 3′-end portion of the zebrafish cDNA by PCR in conjunction with the adapter primer supplied with the kit (Invitrogen). PCR was performed according to the manufacturer's instructions using 10 ng of cDNA template and 1 ng of each primer in a total volume of 50 µl per sample. The samples were subjected to touchdown PCR (2 min at 94°C, 5 cycles of 30 sec at 94°C and 2 min at 72°C, 5 cycles of 30 sec at 94°C and 2 min at 70°C, and 25 cycles of 30 sec at 94°C, 30 sec at 65°C, and 120 sec at 72°C). The amplified DNA fragments were then subcloned into the pCRII-TOPO cloning vector and sequenced. Multiple sequence alignments were carried out using Megalign software (DNAstar, Madison, WI). All parameters were set at default. A full-length zebrafish lamin A cDNA fragment was subcloned into the *Kpn*I and *Bam*HI sites of the pEGFP-C1 Vector (Clontech, Mountain View, CA).

### Whole mount mRNA *in situ* hybridization of embryos

For *in situ* hybridization, digoxigenin-labeled antisense RNA probes were synthesized from the 2.0 kb zebrafish lamin A/C insert which had been directionally subcloned into the pCRII-TOPO vector (Invitrogen) using an *in vitro* transcription kit (Roche Applied Science, Indianapolis, IN). Antisense mRNA was transcribed from the *Xba*I-linearized plasmid using SP6 polymerase, and sense mRNA from the *Kpn*I-linearized plasmid using T7 polymerase as a control. *In situ* hybridization analyses and development of whole-mount zebrafish embryos were performed as previously described [83] [84].

### RNA isolation and RT-PCR analysis for zebrafish lamin A/C

Total RNA was extracted from 24 hpf embryos using TRIzol reagent according to the manufacturer's protocol (Invitrogen). Double-stranded cDNA was synthesized using M-MLV reverse transcriptase (Promega), followed by PCR with Ex*Taq* (Takara). For RT-PCR analysis of zlamin A/C, the forward and reverse primers used were 5′- TCAGCAAAGTGCGTGAGGACTACA-3′ and 5′-GCGAAGCTGCTCTTCATGTTGGTT-3′. Specifically for zlamin A, the forward and reverse primers used were 5′-TTTGTTCCAGACCACCCTCATC-3′ and 5′-AAGCACAGATGGTCAGGGTTTG-3′: and for zlamin C, 5′-TGGCAGGTGAAGAGGCAGATTG-3′ and 5′-GACCCGCAACATAAAGGCTA -3′. For the detection of the cryptic splicing region in lamin A exon 11 caused by MO (z*LMNA*-MO2 and z*LMNA*-MO2.1) injections, the primers used were as follows: forward, 5′-TTTGTTCCAGACCACCCTCATC-3′ and reverse, 5′-AAGCACAGATGGTCAGGGTTTG-3′ (as described above). As for the control primers, the forward and reverse primers used to amplify EF1α were 5′-ACCACCGGCCATCTGATCTACAAA-3′ and 5′-ACGGATGTCCTT GACAGACACGTT -3′, respectively. The forward and reverse primers used to amplify β-actin were 5′-CCCAGACATCAGGGAGTGAT-3′ and 5′-CACCGATCCAGACGGAGTAT-3′, respectively [85]. For amplification by PCR, the initial denaturing step at 94°C for 5 min was followed by 25 amplification cycles of 30 sec at 94°C; 30 sec at 60°C; 60 sec at 72°C, and a final extension period of 10 min at 72°C. Each amplification reaction was separated on a 1.5% agarose gel stained with ethidium bromide-stain and the bands were visualized and recorded using a Multi Image Light Cabinet (Cell Bioscience, Santa Clara, CA).

### Morpholino oligonucleotides

Morpholino oligonucleotides (MOs) were designed and synthesized by Gene Tools, LLC (Philomath, OR), to target the 5′-translation start site (z*LMNA*-MO1) and the eleventh exon-intron junction (z*LMNA*-MO2 and z*LMNA*-MO2.1) of the zebrafish *LMNA* gene. The MO sequences were as follows: z*LMNA*-MO1, 5′-CATGGTTGTCTGGAACTACTGATA-3′; z*LMNA*-MO2, 5′-ACATACAGTACACACCTGTCTGGGT-3′; z*LMNA*-MO2.1; 5′-ACACATACAGTACACACCTGTCTGG-3′; 5-base mismatch control for z*LMNA*-MO1 (MO1-5-mis), 5′-CATGcTTcTgTGcAACTACTcATA-3′ (mispairing bases are indicated in lowercase); 5-base mismatch control for z*LMNA*-MO2 (MO2-5-mis), 5′-ACATAgAcTACAgACCTcTCTcGGT-3′. MOs were re-suspended in sterile water at a concentration of 1 mM as stock solutions. For microinjection into embryos, the stock solutions (1 mM) were diluted to 125, 250, and 500 µM. A 10 nl volume of each MO solution was injected into the yolk during the one-cell stage. For transfection into cultured cells, the stock solution was diluted to 30 µM as a final concentration. Zebrafish AB9 fibroblast cells were cultured in DMEM supplemented with 15% heat-inactivated feral bovine serum (FBS) and grown in a humidified 5% CO_2_-containing incubator at 28.5°C. Endo-Portor (Gene Tools) was used to help cells incorporate z*LMNA* MOs.

### Western blot analysis

The zebrafish cells, embryos, and the tissues of adults were homogenized in RIPA buffer supplemented with one tablet of protease inhibitor cocktail (Roche Applied Science)/10 ml on ice. The protein extracts were then fractionated on 10% SDS-polyacrylamide gels and transferred to PVDF membranes (Bio-Rad, Hercules, CA). The blot was incubated with the primary antibody diluted in TBST (1∶2000 dilution anti-lamin A antibody, ab14309; Abcam, Cambridge, MA) overnight at 4°C. After washing, the blot was then incubated with the secondary anti-IgY antibody (1∶2000 dilution, ab6753; Abcam) at room temperature for 1 h and visualized using an ECL kit (Perkin Elmer, Waltham, MA) in accordance with the manufacturer's instructions. In addition, reactivity with an actin antibody (AC-40; Sigma-Aldrich, St. Louis, MO) at a dilution of 1∶1000 was used to normalize the loading per lane.

### Antibody staining for muscle and Alcian blue staining for cartilage

Antibody staining for muscle of zebrafish embryo was performed as previously described [86]. The following primary antibodies were used for immunostaining: 24 hpf embryos for F59 (1∶100) and 48 hpf embryos for F310 (1∶100) (DSHB, Iowa City, IW). Immunoreactivity was detected using a rhodamine (TRITC)-conjugated anti-mouse secondary antibody (Southern Biotechnology Associates, Birmingham, AL) diluted in blocking solution (PBS containing 1% BSA, 2% Normal goat serum, 0.1% Tween-20, 1% DMSO) (1∶250). Fish larvae at 6 dpf were stained with Alcian blue to visualize cartilage as previously described [87].

### Senescence-associated β-gal activity (SA-β-gal) assay of zebrafish embryos

Zebrafish larvae at 3.5–6 dpf were washed in phosphate buffer saline (PBS) three times and fixed overnight in 4% paraformaldehyde (PFA) with PBS at 4°C. Adult zebrafish were also washed in PBS 3 times and fixed for 3 days in 4% PFA in PBS. After fixation, samples were washed 3 times in PBS, pH 7.5, twice again in PBS, pH 6.0 at 4°C, and then incubated at 37°C (in the absence of CO_2_) for 12–16 h with SA-β-gal staining solution (5 mM potassium ferricyanide, 5 mM potassium ferrocyanide, 2 mM MgCl_2_ in PBS at pH 6.0). Staining intensity was quantified by pixel analysis using with Adobe Photoshop CS software as described previously [42].

### Apoptosis detection and Immunohistochemistry

Embryos at 24–48 hpf, were fixed overnight in 4% PFA with PBS at 4°C and stored in 100% methanol at −20°C until samples were used. Samples were then incubated in 100% acetone at −20°C for 20 min. Following fixation, the embryos were rinsed three times with PBS containing 0.1% Tween-20 (PBST; 5 min each). Samples were then permeabilized by treatment with 0.5% Triton X-100 and 0.1% sodium citrate in PBS for 15 min, then with 5 to 50 mg/ml proteinase K (Invitrogen) for 5 to 25 min depending on embryo stages. Embryos were subjected to TUNEL assay by using the ApopTag Red *in situ* Apoptosis Detection Kit (Chemicon, Temecula, CA) according to the manufacture's instruction.

For whole-mount pH 3 antibody staining, embryos at 24 hpf were fixed overnight in 4% PFA with PBS at 4°C, incubated in 100% methanol at −20°C, and then treated with 100% acetone at −20°C for 7 min. After fixation, embryos were rinsed in 50% PBS/50% methanol at −20°C for 1 h, rinsed once in water and then twice in PBST (5 min each). Embryos were blocked for 1 h in blocking solution (PBS containing 1% BSA, 2% Normal goat serum, 0.1% Tween-20, 1% DMSO) and then incubated overnight at 4°C with a polyclonal anti-pH 3 (Ser10) antibody (Upstate-Millipore, Billerica, MA) (1∶200). Finally, the embryos were washed 4 times in PBS-DT (PBS including, 0.1% Tween-20 and 1% DMSO) for 15 min, and incubated in TRITC-conjugated anti-rabbit secondary antibody (Southern Biotechnology) (1∶300) in blocking solution for 4 h at room temperature.

BrdU (Sigma) with a final concentration of 10 mM was dissolved in E3 medium (5 mM NaCl, 0.17 mM KCl, 0.33 mM CaCl_2_, 0.33 mM MgSO_4_) containing 15% dimethyl sulfoxide (DMSO). Embryos at 24 hpf were soaked in the BrdU solution for 20 min at 6°C, then incubated for 5–10 min at 28.5°C. Samples were fixed for 2 h in 4% PFA with PBS at room temperature, rinsed twice in PBS, incubated in 2 N HCl for 30 min at room temperature, and transferred in 0.1 M Tris-HCl (pH 7.5) for 5 min, blocked in FCS-PBST (10% fetal calf serum, 1% DMSO, in PBST) solution for 30 min, and incubated with anti-BrdU Cy3-labeled antibody (BD-Biosciences-Pharmingen, San Jose, CA) (1∶100).

Anti-PCNA FITC-labeled antibody (BD-Biosciences-Pharmingen,) (1∶100) staining was performed by the same procedure done in pH3 antibody staining as described above, except the secondary antibody.

### Detection and quantitation of visible and fluorescent images

All animals were photographed under the same conditions using reflected light (for SA-β-gal) or fluorescence (for BrdU and pH 3) under a macro microscope, AZ100 (Nikon). SA-β-gal activity in each animal was quantitated using a selection tool in Adobe Photoshop for a color range that was chosen by 25 additive blue color selections of regions that showed visually positive SA-β-gal staining. For analyses of embryos, these regions were selected in each embryo proper only and not in the yolk. This was to eliminate variability due to differences in initial yolk volume and yolk consumption over time. Since the yolk stains much more intensely blue for SA-β-gal at all stages of development than any other embryonic tissues, it was desirable to exclude this as a source of variability. Following pixel selection, a fuzziness setting of 14 was used, and the chosen pixel number was calculated using the image histogram calculation. Incorporated BrdU and pH 3 positive cells in each animal were quantitated in the same way except that a fuzziness setting of 64 was used.

### Oil red O staining

Zebrafish larvae at 5 dpf were PBS three times and fixed overnight in 4% PFA with PBS at 4°C. After fixation, samples were washed twice in PBS, followed by oil red O staining in freshly filtered 0.3% oil red O in 60% 2-propanol for 30 min to 2 h [54]. Samples were quickly washed in 60% 2-propanol for a few times, and then finally washed once in PBS.

### Semi-quantitative RT-PCR

First strand cDNA was generated using total RNA that had been isolated from *LMNA* MO- and 5-base mismatch control (5-mis) MO-injected 24 hpf embryos with Trizol (Invitrogen). The linear amplification ranges were then determined for each of the primer sets as described previously [85]. Primers used to amplify the zebrafish cdk2 and PPARγ genes were designed using Genetyx mac software (cdk2, forward primer: 5′-AGTTACAGGAGAAACTGTCGCGCT-3′, reverse primer: 5′-AGCGTTCTTTGCCGAGATCCTCTT-3′; PPARγ, forward primer: 5′-AAGAGCCGCAACAAGTGCCAATAC-3′; reverse primer: 5′-AGAACTCGAACTTGGGCTCCATCA-3′). All other primer sequences had been reported previously by Liu et al. [85]. PCR conditions were as follows: an initial denaturing step at 94°C for 5 min was followed by 25 amplification cycles of 30 sec at 94°C; 30 sec at 60°C; 60 sec at 72°C, and a final extension period of 10 min at 72°C.

### Generating transgenic zebrafish

To generate transgenic zebrafish expressing EGFP-tagged zProgerin/zlamin A-Δ37, the corresponding expression construct was generated using the QuikChange Site-Directed Mutagenesis Kit (Stratagene, La Jolla, CA) in accordance with the manufacturer's instructions. This vector was then injected into one-cell stage embryos with meganuclease I*-Sce*I [88] [89]. Germline transgenic founders (F_0_) were identified by screening F_1_ embryos via PCR. Four founder transgenic fish carrying the EGFP-zProgerin DNA construct were thus identified. F_1_ embryos of these transgenic founders were then allowed to grow to maturity. Adult transgenic fish of the F_1_ generation were screened individually by PCR using DNA extracted from their caudal fins. For genotyping, EGFP-specific primers (EGFP-sense: 5′-TCTTCTTCAAGGACGACGGCAACT-3′ and EGFP-antisense: 5′-TGTGGCGGATCTTGAAGTTCACCT-3′) and control PCR primers (Wnt5a-sense: 5′-CAGTTCTCACGTCTGCTACTTGCA-3′ and Wnt5a-antisense: 5′-ACTTCCGGCGTGTTGGAGATTTC-3′) were mixed and 1 µM of each was used in a PCR reaction with a final volume of 25 µl.

### Drug treatments

The farnesyltransferase inhibitor FTI L-744832 (Biomol, Plymouth Meeting, PA) was resuspended in DMSO (50 mM). For FTI treatment, cells were cultured to 70% confluence, and treated with 5, 10, and 20 µM of the inhibitor in cell culture medium for 72 h until harvesting. Embryo FTI treatments (20, 40, and 100 µM) were performed from 5 through 30 hpf in E3 medium with 1% DMSO at 28.5°C.

### Quantitative analysis and statistics

Data processing and statistical analyses were performed using Statistical Package for the Social Sciences (SPSS) version 14.0. This software was used to generate each of the scatter plots, tables and graphs shown in the text and perform statistical tests where appropriate. Additional statistical analyses were performed using a custom MATLAB script to analyze survival estimates by the method of Kaplan and Meier, and to compare of survival between mutant fish and their wild type controls using the log rank test [90].

## Supporting Information

Figure S1 **The sequence and genetic organization of zebrafish lamin A/C is conserved with human lamin A/C.** The zebrafish lamin A/C gene (zlamin A/C) comprises 12 exons, encoding two globular domains and a rod domain; zlamin C is encoded by exons 1 to 9.
(TIF)

Figure S2 **Expression of zebrafish lamin A/C in adult fish.** Immunofluorescence microscopy of 1-year old adult zebrafish fin cells *in situ*. The immunostaining reveals that zebrafish lamin A/C is localized to the nuclear envelope.
(TIF)

Figure S3 **Expression of zebrafish lamin A/C during early development.** (**A**) RT-PCR analysis of zlamin A/C, zlamin A and zlamin C during early development. (**B**) Expression patterns of zlamin A/C during early development (0.75 to 72 hpf) (a–e, g, i, k, and n are lateral views; f, h, j, m, and p are dorsal views; l, and o are ventral views. pf: pectoral fin; op: opercle).
(TIF)

Figure S4 **Schematic representation of the nucleotide sequences targeted by z** ***LMNA*** **-MO2 and z** ***LMNA*** **-MO2.1.** The nucleotide sequences of exon 10 (Ex. 10), intron between Ex.10 and exon 11 (Ex. 11), Ex. 11, and intron between Ex.11 and exon 12 (Ex. 12) are shown with the annealing portions of z*LMNA*-MO2 (MO2) and z*LMNA*-MO2.1 (MO2.1).
(TIF)

Figure S5 **MO-injected embryos stained with anti-PCNA.** Lateral views of MO-injected embryos at 24 hpf stained with the PCNA antibody.
(TIF)

Figure S6 **PPARγ mRNA expression in lamin A/C morphants.** The expression of zebrafish PPARγ in the morphants was semi-quantitatively analyzed by single-embryo RT-PCR at 72 hpf. Two independent samples (lanes 1 and 2) for MO1 (both 5-mis and MO) and MO2 (both 5-mis and MO) were loaded in the lanes (upper picture panels). In quantitation (lower graph panel), the expression levels of PPARγ mRNA were significantly decreased in z*LMNA*-MO-injected (MO) embryos in comparison with the controls (5-mis) (*P*<0.01 for 5-mis versus MO in MO1 and MO2).
(TIF)

Data S1 (DOC)
