# Supplementary figures and images for: Embryonic Senescence and Laminopathies in a Progeroid Zebrafish Model
Source: PLoS One. 2011 Mar 30;6(3):e17688. doi: 10.1371/journal.pone.0017688 (PMC3068137; doi:10.1371/journal.pone.0017688)

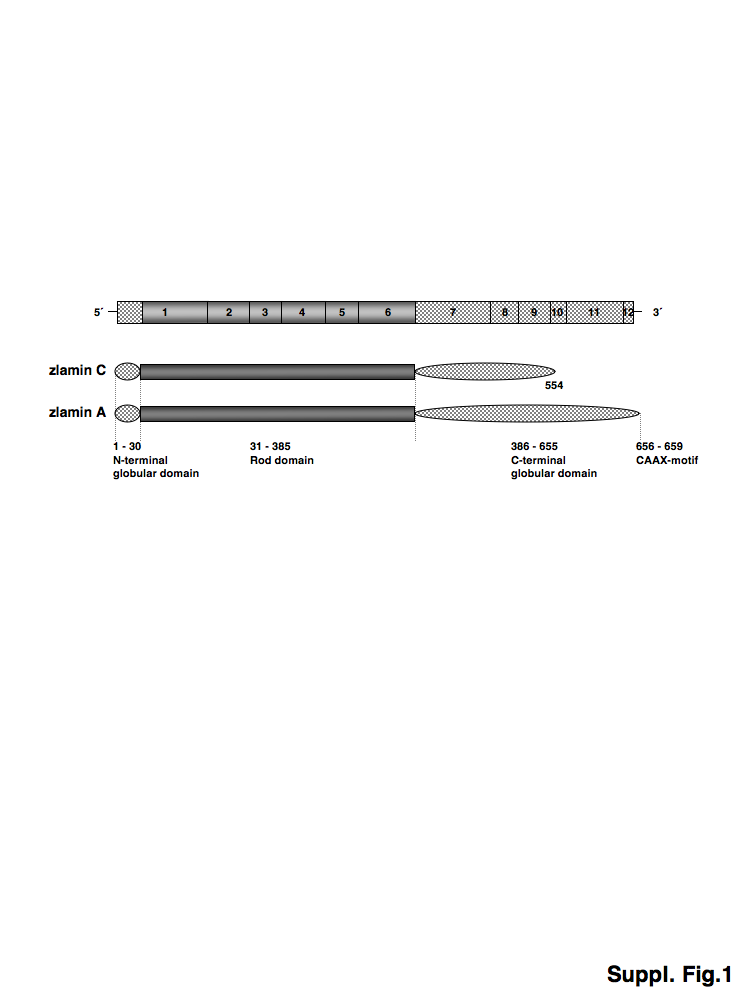

Supplement: Figure S1 — The sequence and genetic organization of zebrafish lamin A/C is conserved with human lamin A/C. The zebrafish lamin A/C gene (zlamin A/C) comprises 12 exons, encoding two globular domains and a rod domain; zlamin C is encoded by exons 1 to 9. (TIF) [file pone.0017688.s001.tif]

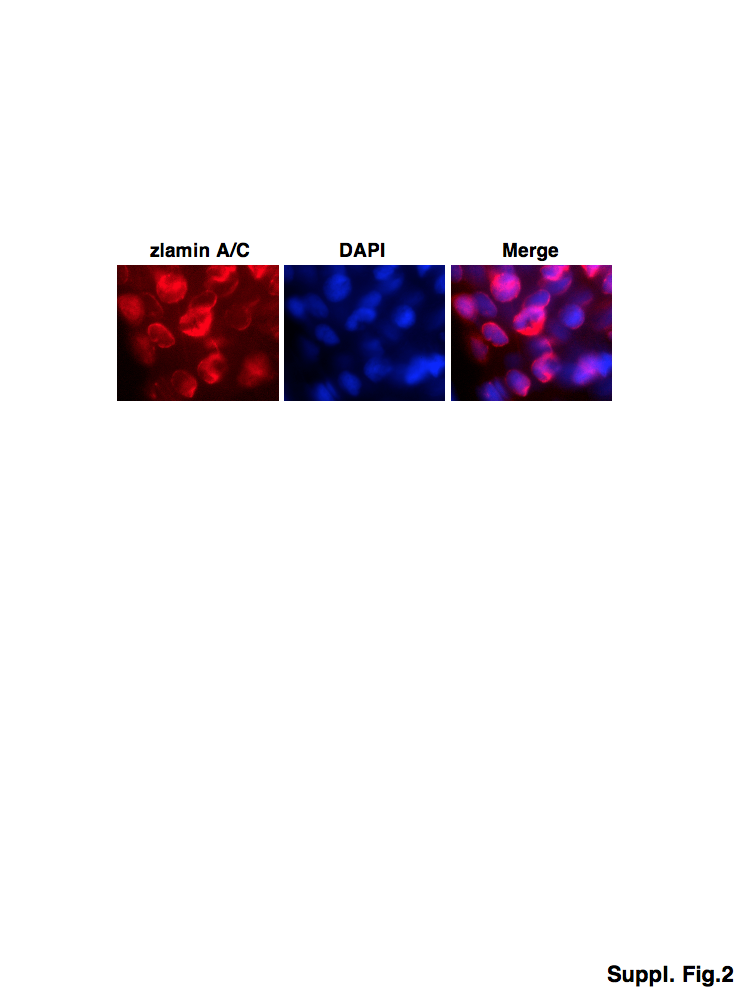

Supplement: Figure S2 — Expression of zebrafish lamin A/C in adult fish. Immunofluorescence microscopy of 1-year old adult zebrafish fin cells in situ. The immunostaining reveals that zebrafish lamin A/C is localized to the nuclear envelope. (TIF) [file pone.0017688.s002.tif]

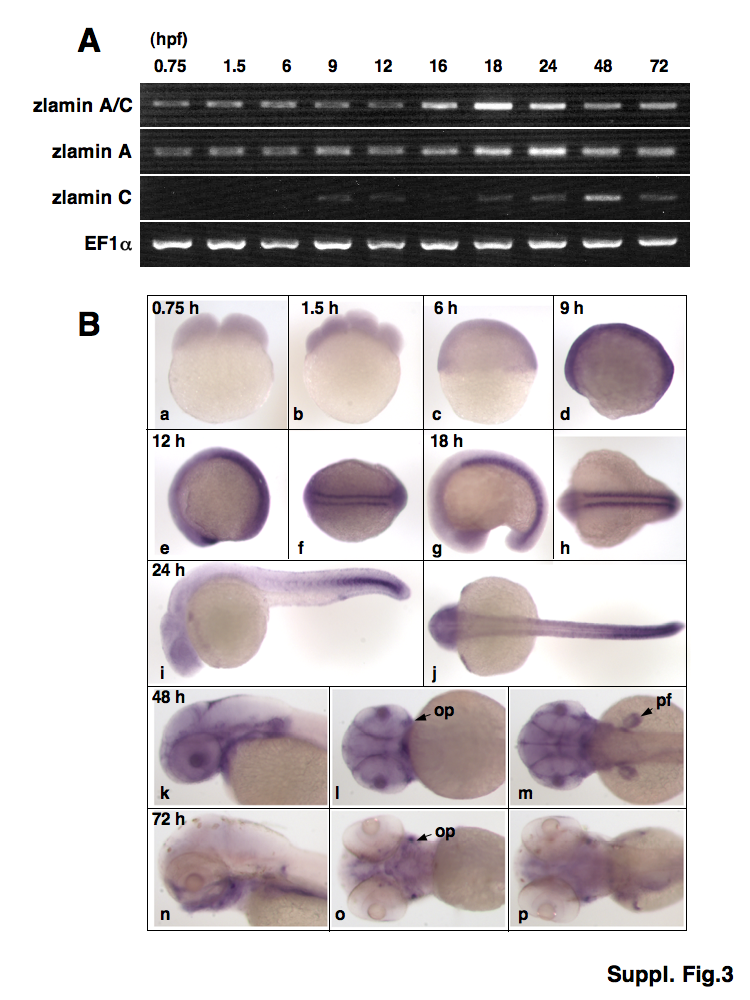

Supplement: Figure S3 — Expression of zebrafish lamin A/C during early development. (A) RT-PCR analysis of zlamin A/C, zlamin A and zlamin C during early development. (B) Expression patterns of zlamin A/C during early development (0.75 to 72 hpf) (a–e, g, i, k, and n are lateral views; f, h, j, m, and p are dorsal views; l, and o are ventral views. pf: pectoral fin; op: opercle). (TIF) [file pone.0017688.s003.tif]

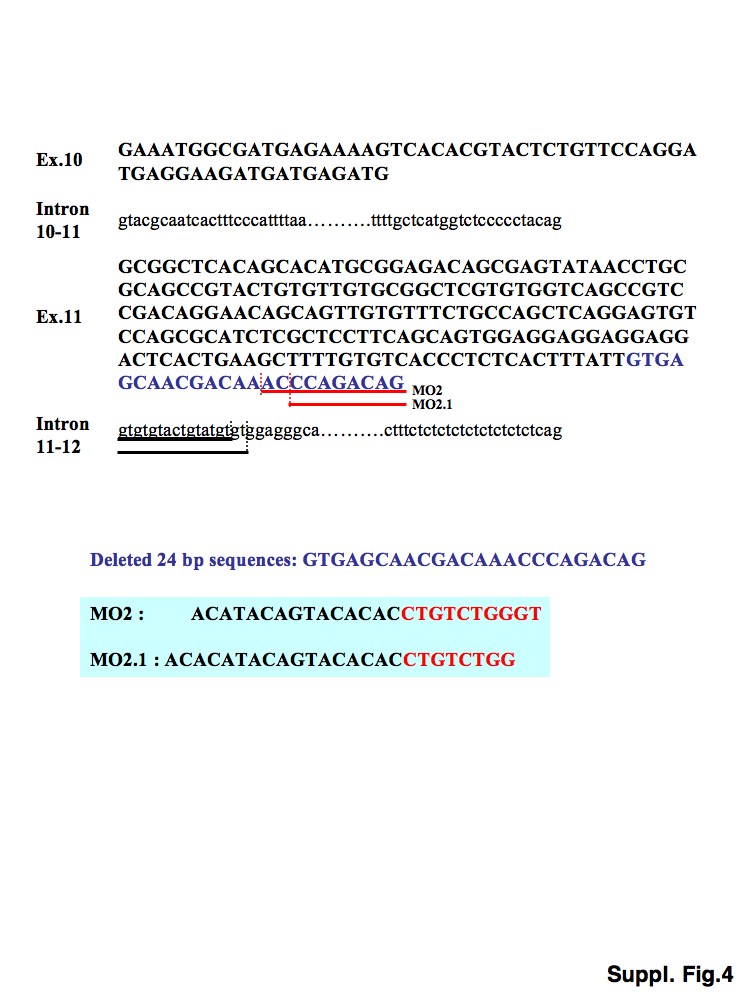

Supplement: Figure S4 — Schematic representation of the nucleotide sequences targeted by z LMNA -MO2 and z LMNA -MO2.1. The nucleotide sequences of exon 10 (Ex. 10), intron between Ex.10 and exon 11 (Ex. 11), Ex. 11, and intron between Ex.11 and exon 12 (Ex. 12) are shown with the annealing portions of zLMNA-MO2 (MO2) and zLMNA-MO2.1 (MO2.1). (TIF) [file pone.0017688.s004.tif]

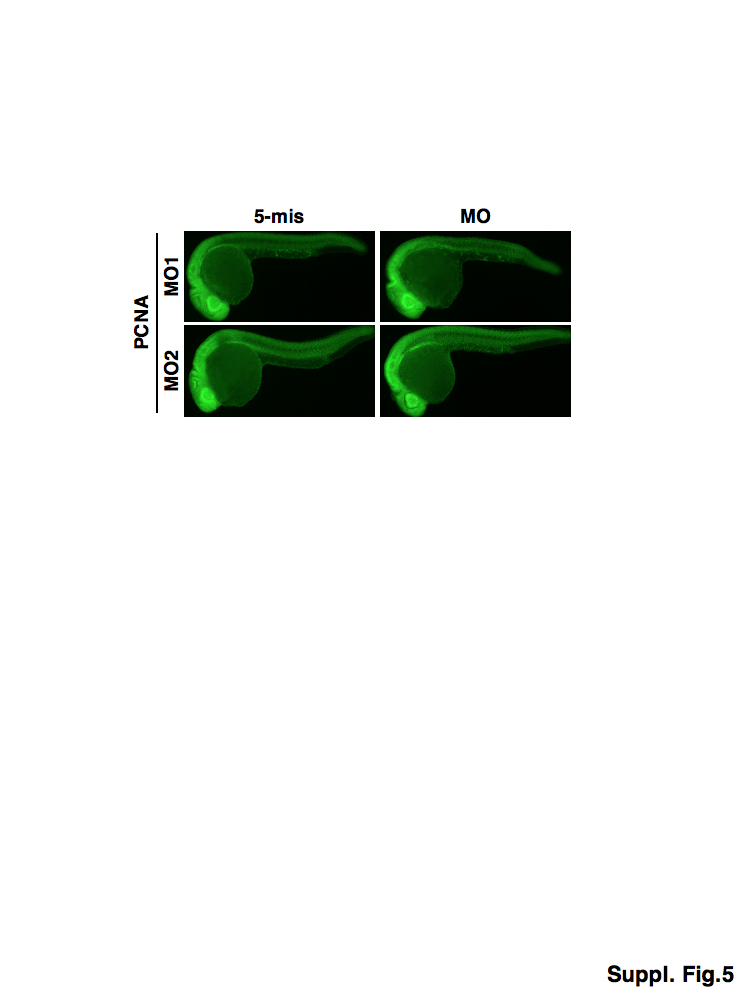

Supplement: Figure S5 — MO-injected embryos stained with anti-PCNA. Lateral views of MO-injected embryos at 24 hpf stained with the PCNA antibody. (TIF) [file pone.0017688.s005.tif]

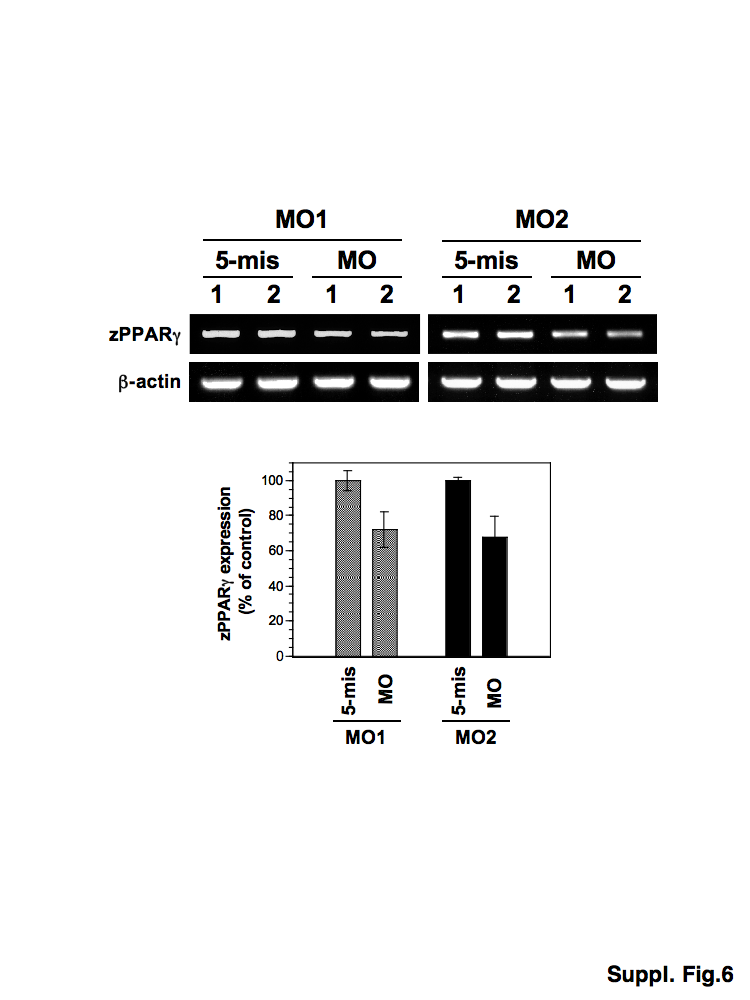

Supplement: Figure S6 — PPARγ mRNA expression in lamin A/C morphants. The expression of zebrafish PPARγ in the morphants was semi-quantitatively analyzed by single-embryo RT-PCR at 72 hpf. Two independent samples (lanes 1 and 2) for MO1 (both 5-mis and MO) and MO2 (both 5-mis and MO) were loaded in the lanes (upper picture panels). In quantitation (lower graph panel), the expression levels of PPARγ mRNA were significantly decreased in zLMNA-MO-injected (MO) embryos in comparison with the controls (5-mis) (P<0.01 for 5-mis versus MO in MO1 and MO2). (TIF) [file pone.0017688.s006.tif]
