# Supplementary material for: Embryonic Senescence and Laminopathies in a Progeroid Zebrafish Model
Source: PLoS One. 2011 Mar 30;6(3):e17688. doi: 10.1371/journal.pone.0017688 (PMC3068137; doi:10.1371/journal.pone.0017688)
Supplement: Data S1 — (DOC) [file pone.0017688.s007.doc]

**Supplemental Data**

***Expression of Lamin A/C in Adult Zebrafish and During Early Development***

In addition to RT-PCR (**Fig. 1B, upper panel**) and western blot analyses (**Fig. 1B, lower panels**) of zlamin A/C expressions in adult tissues, we also detected the intrinsic zlamin A/C visualized by immunostaining in the adult fin and this analysis showed a nuclear envelope localization (**Suppl.** **Fig. 2**). On the other hand, zlamin A/C protein expression was undetectable by the same antibody during early development to 5 days after fertilization. To confirm whether lamin A/C was expressed during early development, we also performed both RT-PCR and western blotting in samples from embryos. RT-PCR analysis was used to semi-quantify the transcript levels of the three different isoforms of zlamin A/C. The expression of zlamin A/C mRNA increased during the 16 to 24 hpf period with a peak at 18 hpf, followed by a decrease at 72 hpf. Lamin C is an alternatively spliced form of lamin A [1]. To confirm any differences between the expression of zlamin A and zlamin C, we designed specific primers (see Materials and Methods) and found that the zlamin A expression profile was similar to that of zlamin A/C. zLamin C was detectable from 9 to 72 hpf, and had a stronger signal at 48 hpf (**Fig. S3A**). The mRNA levels of zlamin A were constantly higher than those of zlamin C throughout the early embryonic stages (**Suppl.** **Fig. 3A**).

We next attempted to detect the expression of zlamin A/C in embryos by whole mount *in situ* hybridization assays using digoxigenin-labeled sense and antisense RNA probes against zlamin A/C, zlamin A and C (see Materials and Methods). Some maternally provided lamin A/C mRNA was detectable prior to the midblastula transition (MBT) (**Fig. S3B**). As a general time course, an increase in intrinsic zlamin A/C mRNA expression was clearly detectable by 9 hpf. At 18 hpf, it was expressed in the entire population of adaxial cells, later more specifically in the caudal adaxial cells (some caudal somites) by 24-30 hpf, and had disappeared by 48 hpf. At 48 hpf, the expression of zlamin A/C was observed in the head region, particularly the developing pectoral fins and opercle. At 72 hpf, its expression was observed in the opercle and ceratohyal. Although the expression of zlamin C was detectable by RT-PCR, zlamin C-specific mRNA was not detected in the zebrafish embryos by *in situ* hybridization, which is likely due to the AT-rich region in the zlamin C-specific sequence which would reduce the efficiency of probe hybridization. Of note, no specific staining was detectable using a sense control RNA probe at any developmental time point tested (data not shown).

**References**

1. Lin F, Worman HJ (1993) Structural organization of the human gene encoding nuclear lamin A and nuclear lamin C. J Biol Chem 268: 16321-16326.
